# Toxicity, mechanism and health effects of some heavy metals

**DOI:** 10.2478/intox-2014-0009

**Published:** 2014-11-15

**Authors:** Monisha Jaishankar, Tenzin Tseten, Naresh Anbalagan, Blessy B. Mathew, Krishnamurthy N. Beeregowda

**Affiliations:** Department of Biotechnology, Sapthagiri College of Engineering, Bangalore-57, Karnataka, India

**Keywords:** heavy metals, metal toxicity, oxidative stress, free radicals

## Abstract

Heavy metal toxicity has proven to be a major threat and there are several health risks associated with it. The toxic effects of these metals, even though they do not have any biological role, remain present in some or the other form harmful for the human body and its proper functioning. They sometimes act as a pseudo element of the body while at certain times they may even interfere with metabolic processes. Few metals, such as aluminium, can be removed through elimination activities, while some metals get accumulated in the body and food chain, exhibiting a chronic nature. Various public health measures have been undertaken to control, prevent and treat metal toxicity occurring at various levels, such as occupational exposure, accidents and environmental factors. Metal toxicity depends upon the absorbed dose, the route of exposure and duration of exposure, i.e. acute or chronic. This can lead to various disorders and can also result in excessive damage due to oxidative stress induced by free radical formation. This review gives details about some heavy metals and their toxicity mechanisms, along with their health effects.

## Introduction

**Figure 1:**
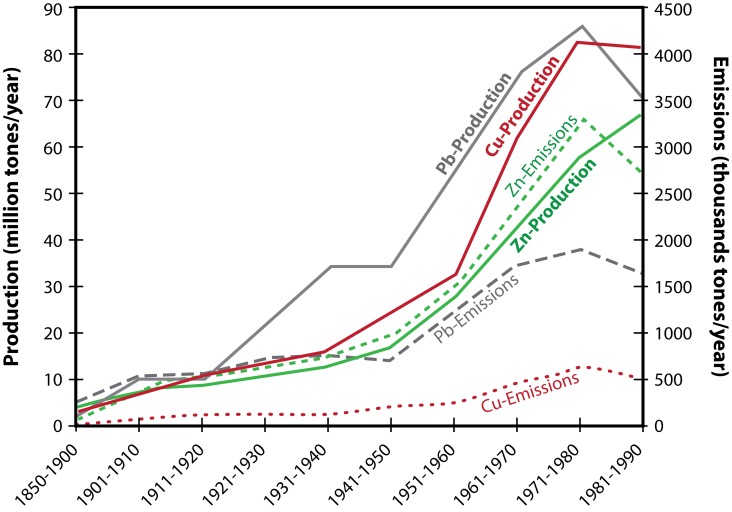
The global production and consumption of selected toxic metals during 1850–1990 (Adapted from Nriagu, 1996).

Metals are substances with high electrical conductivity, malleability, and luster, which voluntarily lose their electrons to form cations. Metals are found naturally in the earth's crust and their compositions vary among different localities, resulting in spatial variations of surrounding concentrations. The metal distribution in the atmosphere is monitored by the properties of the given metal and by various environmental factors (Khlifi & Hamza-Chaffai, 2010). The main objective of this review is to provide insight into the sources of heavy metals and their harmful effects on the environment and living organisms. Heavy metals are generally referred to as those metals which possess a specific density of more than 5 g/cm^3^ and adversely affect the environment and living organisms (Järup, 2003). These metals are quintessential to maintain various biochemical and physiological functions in living organisms when in very low concentrations, however they become noxious when they exceed certain threshold concentrations. Although it is acknowledged that heavy metals have many adverse health effects and last for a long period of time, heavy metal exposure continues and is increasing in many parts of the world. Heavy metals are significant environmental pollutants and their toxicity is a problem of increasing significance for ecological, evolutionary, nutritional and environmental reasons (Jaishankar *et al.*, 2013; Nagajyoti *et al.*, 2010). The most commonly found heavy metals in waste water include arsenic, cadmium, chromium, copper, lead, nickel, and zinc, all of which cause risks for human health and the environment (Lambert *et al.*, 2000). Heavy metals enter the surroundings by natural means and through human activities. Various sources of heavy metals include soil erosion, natural weathering of the earth's crust, mining, industrial effluents, urban runoff, sewage discharge, insect or disease control agents applied to crops, and many others (Morais *et al.*, 2012). Figure 1 shows the global production and consumption of selected toxic metals during 1850–1990 (Nriagu, 1996).

Although these metals have crucial biological functions in plants and animals, sometimes their chemical coordination and oxidation-reduction properties have given them an additional benefit so that they can escape control mechanisms such as homeostasis, transport, compartmentalization and binding to required cell constituents. These metals bind with protein sites which are not made for them by displacing original metals from their natural binding sites causing malfunctioning of cells and ultimately toxicity. Previous research has found that oxidative deterioration of biological macromolecules is primarily due to binding of heavy metals to the DNA and nuclear proteins (Flora *et al.*, 2008).

## Heavy metals and their toxicity mechanisms

### Arsenic

Arsenic is one of the most important heavy metals causing disquiet from both ecological and individual health standpoints (Hughes *et al.*, 1988). It has a semimetallic property, is prominently toxic and carcinogenic, and is extensively available in the form of oxides or sulfides or as a salt of iron, sodium, calcium, copper, *etc.* (Singh *et al.*, 2007). Arsenic is the twentieth most abundant element on earth and its inorganic forms such as arsenite and arsenate compounds are lethal to the environment and living creatures. Humans may encounter arsenic by natural means, industrial source, or from unintended sources. Drinking water may get contaminated by use of arsenical pesticides, natural mineral deposits or inappropriate disposal of arsenical chemicals. Deliberate consumption of arsenic in case of suicidal attempts or accidental consumption by children may also result in cases of acute poisoning (Mazumder, 2008; Saha *et al.*, 1999). Arsenic is a protoplastic poison since it affects primarily the sulphydryl group of cells causing malfunctioning of cell respiration, cell enzymes and mitosis (Gordon & Quastel, 1948).

#### Mechanism of arsenic toxicity

In arsenic biotransformation, harmful inorganic arsenic compounds get methylated by bacteria, algae, fungi and humans to give monomethylarsonic acid (MMA) and dimethylarsinic acid (DMA). In this biotransformation process, these inorganic arsenic species (iAs) are converted enzymetically to methylated arsenicals which are the end metabolites and the biomarker of chronic arsenic exposure.

iAs(V)→iAs(III)→MMA(V)→MMA(III)→DMA(V)

Biomethylation is a detoxification process and end products are methylated inorganic arsenic such as MMA (V) and DMA (V), which excreted through urine are bioindication of chronic arsenic exposure. However MMA (III) is not excreted and remains inside the cell as an intermediate product.

Monomethylarsonic acid (MMA III), an intermediate product, is found to be highly toxic compared to other arsenicals, potentially accountable for arsenic-induced carcinogenesis (Singh *et al.*, 2007).

### Lead

**Figure 2:**
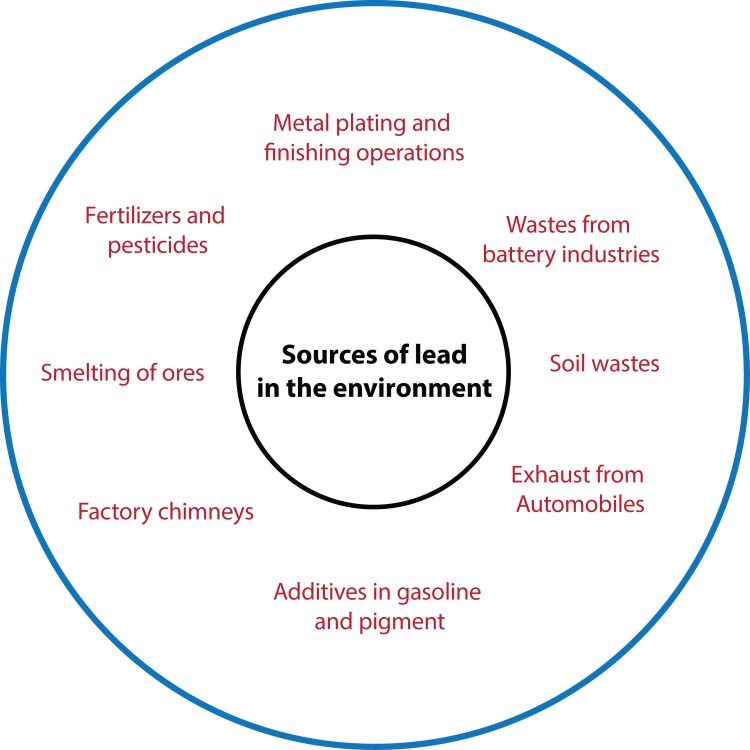
Various sources of lead pollution in the environment (Adapted from Sharma & Dubey, 2005).

Lead is a highly toxic metal whose widespread use has caused extensive environmental contamination and health problems in many parts of the world. Lead is a bright silvery metal, slightly bluish in a dry atmosphere. It begins to tarnish on contact with air, thereby forming a complex mixture of compounds, depending on the given conditions. Figure 2 shows various sources of lead pollution in the environment (Sharma & Dubey, 2005). The sources of lead exposure include mainly industrial processes, food and smoking, drinking water and domestic sources. The sources of lead were gasoline and house paint, which has been extended to lead bullets, plumbing pipes, pewter pitchers, storage batteries, toys and faucets (Thürmer *et al.*, 2002). In the US, more than 100 to 200,000 tons of lead per year is being released from vehicle exhausts. Some is taken up by plants, fixation to soil and flow into water bodies, hence human exposure of lead in the general population is either due to food or drinking water (Goyer, 1990). Lead is an extremely toxic heavy metal that disturbs various plant physiological processes and unlike other metals, such as zinc, copper and manganese, it does not play any biological functions. A plant with high lead concentration fastens the production of reactive oxygen species (ROS), causing lipid membrane damage that ultimately leads to damage of chlorophyll and photosynthetic processes and suppresses the overall growth of the plant (Najeeb *et al.*, 2014). Some research revealed that lead is capable of inhibiting the growth of tea plant by reducing biomass and debases the tea quality by changing the quality of its components (Yongsheng *et al.*, 2011). Even at low concentrations, lead treatment was found to cause huge instability in ion uptake by plants, which in turn leads to significant metabolic changes in photosynthetic capacity and ultimately in a strong inhibition of plant growth (Mostafa *et al.*, 2012).

#### Mechanisms of lead toxicity

**Figure 3:**
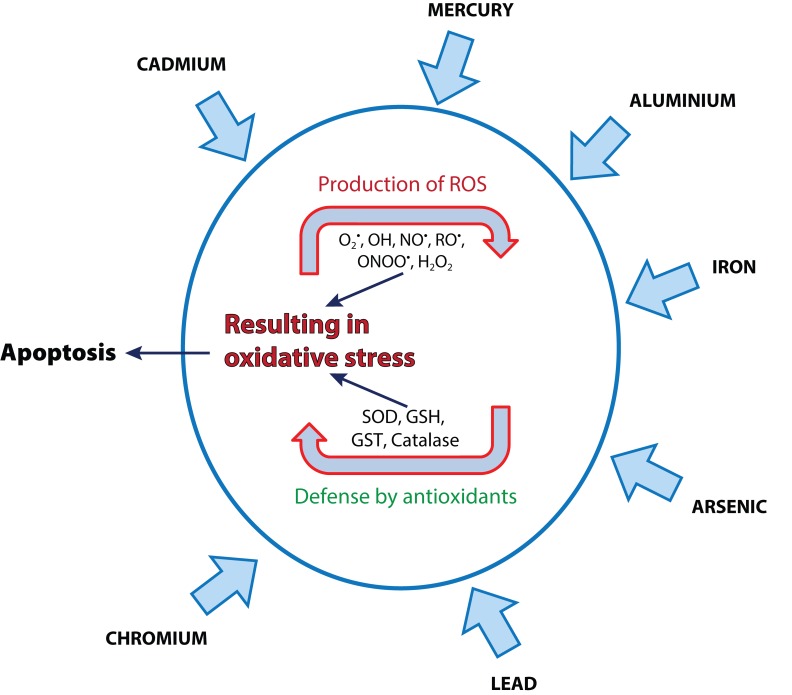
The attack of heavy metals on a cell and the balance between ROS production and the subsequent defense presented by antioxidants.

Lead metal causes toxicity in living cells by following ionic mechanism and that of oxidative stress. Many researchers have shown that oxidative stress in living cells is caused by the imbalance between the production of free radicals and the generation of antioxidants to detoxify the reactive intermediates or to repair the resulting damage. Figure 3 shows the attack of heavy metals on a cell and the balance between ROS production and the subsequent defense presented by antioxidants. Antioxidants, as *e.g.* glutathione, present in the cell protect it from free radicals such as H_2_O_2._ Under the influence of lead, however, the level of the ROS increases and the level of antioxidants decreases. Since glutathione exists both in reduced (GSH) and oxidized (GSSG) state, the reduced form of glutathione gives its reducing equivalents (H^+^ + e^−^) from its thiol groups of cystein to ROS in order to make them stable. In the presence of the enzyme glutathione peroxidase, reduced glutathione readily binds with another molecule of glutathione after donating the electron and forms glutathione disulfide (GSSG). The reduced form (GSH) of glutathione accounts for 90% of the total glutathione content and the oxidized form (GSSG) accounts for 10% under normal conditions. Yet under the condition of oxidative stress, the concentration of GSSG exceeds the concentration of GSH. Another biomarker for oxidative stress is lipid peroxidation, since the free radical collects electron from lipid molecules present inside the cell membrane, which eventually causes lipid peroxidation (Wadhwa *et al.*, 2012; Flora *et al.*, 2012). At very high concentrations, ROS may cause structural damage to cells, proteins, nucleic acid, membranes and lipids, resulting in a stressed situation at cellular level (Mathew *et al.,* 2011).

The ionic mechanism of lead toxicity occurs mainly due to the ability of lead metal ions to replace other bivalent cations like Ca^2+^, Mg^2+^, Fe^2+^ and monovalent cations like Na^+,^ which ultimately disturbs the biological metabolism of the cell. The ionic mechanism of lead toxicity causes significant changes in various biological processes such as cell adhesion, intra- and inter-cellular signaling, protein folding, maturation, apoptosis, ionic transportation, enzyme regulation, and release of neurotransmitters. Lead can substitute calcium even in picomolar concentration affecting protein kinase C, which regulates neural excitation and memory storage (Flora *et al.*, 2012).

### Mercury

The metallic mercury is a naturally occurring metal which is a shiny silver-white, odorless liquid and becomes colorless and odorless gas when heated. Mercury is very toxic and exceedingly bioaccumulative. Its presence adversely affects the marine environment and hence many studies are directed towards the distribution of mercury in water environment. Major sources of mercury pollution include anthropogenic activities such as agriculture, municipal wastewater discharges, mining, incineration, and discharges of industrial wastewater (Chen *et al.*, 2012).

**Figure 4:**
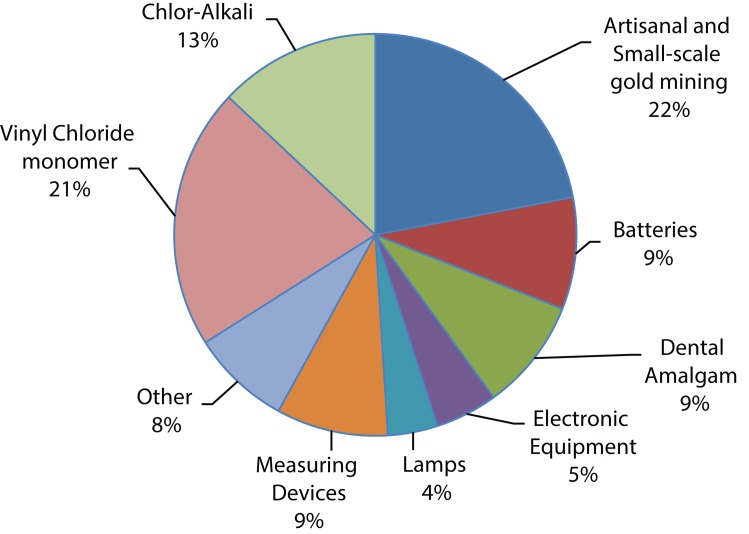
The global usage of mercury for various applications (total in 2005: 3,760 metric tons).

Mercury exists mainly in three forms: metallic elements, inorganic salts and organic compounds, each of which possesses different toxicity and bioavailability. These forms of mercury are present widely in water resources such as lakes, rivers and oceans where they are taken up by the microorganisms and get transformed into methyl mercury within the microorganism, eventually undergoing biomagnification causing significant disturbance to aquatic lives. Consumption of this contaminated aquatic animal is the major route of human exposure to methyl mercury (Trasande *et al*., 2005). Mercury is extensively used in thermometers, barometers, pyrometers, hydrometers, mercury arc lamps, fluorescent lamps and as a catalyst. It is also being used in pulp and paper industries, as a component of batteries and in dental preparations such as amalgams. Figure 4 shows the global usage of mercury for various applications (the GEF and Mercury: The Challenge *By Ibrahima Sow, GEF Climate & Chemicals Team*. Available from: http://www.thegef.org/gef/greenline/april-2012/gef-and-mercury-challenge).

#### Mechanism of mercury toxicity

Mercury is well known as a hazardous metal and its toxicity is a common cause of acute heavy metal poisoning with cases of 3,596 in 1997 by the American Association of Poison Control Centers. Methylmercury is a neurotoxic compound which is responsible for microtubule destruction, mitochondrial damage, lipid peroxidation and accumulation of neurotoxic molecules such as serotonin, aspartate, and glutamate (Patrick, 2002). The total amount of mercury emission into the environment has been assessed at 2,200 metric tons annually (Ferrara *et al.*, 2000). It is estimated that 8 to 10% of American women have mercury levels that would induce neurological disorders in any child they gave birth to, according to both the Environmental Protection Agency and National Academy of Science (Haley, 2005). Animals which are exposed to toxic mercury have shown adverse neurological and behavioral changes. Rabbits when exposed to 28.8 mg/m^3^ mercury vapor for 1 to 13 weeks have shown vague pathological changes, marked cellular degeneration and brain necrosis (Ashe *et al.*, 1953).

The brain remains the target organ for mercury, yet it can impair any organ and lead to malfunctioning of nerves, kidneys and muscles. It can cause disruption to the membrane potential and interrupt with intracellular calcium homeostasis. Mercury binds to freely available thiols as the stability constants are high (Patrick, 2002). Mercury vapors can cause bronchitis, asthma and temporary respiratory problems. Mercury plays a key role in damaging the tertiary and quaternary protein structure and alters the cellular function by attaching to the selenohydryl and sulfhydryl groups which undergo reaction with methyl mercury and hamper the cellular structure. It also intervenes with the process of transcription and translation resulting in the disappearance of ribosomes and eradication of endoplasmic reticulum and the activity of natural killer cells. The cellular integrity is also affected causing free radical formation. The basis for heavy metal chelation is that even though the mercury sulfhydryl bond is stable and divided to surrounding sulfhydryl consisting ligands, it also contributes free sulfhydryl groups to promote metal mobility within the ligands (Bernhoft, 2011).

### Cadmium

Cadmium is the seventh most toxic heavy metal as per ATSDR ranking. It is a by-product of zinc production which humans or animals may get exposed to at work or in the environment. Once this metal gets absorbed by humans, it will accumulate inside the body throughout life. This metal was first used in World War I as a substitute for tin and in paint industries as a pigment. In today's scenario, it is also being used in rechargeable batteries, for special alloys production and also present in tobacco smoke. About three-fourths of cadmium is used in alkaline batteries as an electrode component, the remaining part is used in coatings, pigments and platings and as a plastic stabilizer. Humans may get exposed to this metal primarily by inhalation and ingestion and can suffer from acute and chronic intoxications. Cadmium distributed in the environment will remain in soils and sediments for several decades. Plants gradually take up these metals which get accumulated in them and concentrate along the food chain, reaching ultimately the human body. In the US, more than 500,000 workers get exposed to toxic cadmium each year as per The Agency for Toxic Substances and Disease Registry (Bernard, 2008; Mutlu *et al.*, 2012). Researches have shown that in China the total area polluted by cadmium is more than 11,000 hectares and its annual amount of industrial waste of cadmium discharged into the environment is assessed to be more than 680 tons. In Japan and China, environmental cadmium exposure is comparatively higher than in any other country (Han *et al.*, 2009). Cadmium is predominantly found in fruits and vegetables due to its high rate of soil-to-plant transfer (Satarug *et al.*, 2011). Cadmium is a highly toxic nonessential heavy metal that is well recognized for its adverse influence on the enzymatic systems of cells, oxidative stress and for inducing nutritional deficiency in plants (Irfan *et al.*, 2013).

#### Mechanism of cadmium toxicity

The mechanism of cadmium toxicity is not understood clearly but its effects on cells are known (Patrick, 2003). Cadmium concentration increases 3,000 fold when it binds to cystein-rich protein such as metallothionein. In the liver, the cystein-metallothionein complex causes hepatotoxicity and then it circulates to the kidney and gets accumulated in the renal tissue causing nephrotoxicity. Cadmium has the capability to bind with cystein, glutamate, histidine and aspartate ligands and can lead to the deficiency of iron (Castagnetto *et al.*, 2002). Cadmium and zinc have the same oxidation states and hence cadmium can replace zinc present in metallothionein, thereby inhibiting it from acting as a free radical scavenger within the cell.

### Chromium

Chromium is the seventh most abundant element on earth (Mohanty & Kumar Patra, 2013). Chromium occurs in several oxidation states in the environment ranging from Cr^2+^ to Cr^6+^ (Rodríguez *et al.*, 2009)_._ The most commonly occurring forms of Cr are trivalent- Cr^+3^ and hexavalent- Cr^+6^_,_ with both states being toxic to animals, humans and plants (Mohanty & Kumar Patra, 2013). Chromium occurs naturally by the burning of oil and coal, petroleum from ferro cromate refractory material, pigment oxidants, catalyst, chromium steel, fertilizers, oil well drilling and metal plating tanneries. Anthropogenically, chromium is released into the environment through sewage and fertilizers (Ghani, 2011). Cr(III) is immobile in its reduced form and is insoluble in water whereas Cr(VI) in its oxidized state is highly soluble in water and thus mobile (Wolińska *et al.*, 2013). In order to determine the activities of the metal ions in the environment, metal speciation is very important where in case of chromium the oxidative form of Cr(III) is not an essential contaminant of the ground water but Cr(VI) has been found to be toxic for humans (Gürkan *et al.*, 2012). Cr(III) resides in the organic matter of soil and aquatic environment in the form of oxides, hydroxides and sulphates (Cervantes *et al.*, 2001). Chromium is extensively used in industries such as metallurgy, electroplating, production of paints and pigments, tanning, wood preservation, chemical production and pulp and paper production. These industries play a major role in chromium pollution with an adverse effect on biological and ecological species (Ghani, 2011). A wide range of industrial and agricultural practices increase the toxic level in the environment causing concern about the pollution caused by chromium. Pollution of the environment by chromium, particularly hexavalent chromium, has been the greatest concern in recent years (Zayed & Terry, 2003). Tanneries discharge numerous polluting heavy metals and compounds into the water streams (Nath *et al.*, 2008). Due to the presence of excess oxygen in the environment, Cr (III) is oxidized to Cr (VI), which is extremely toxic and highly soluble in water (Cervantes *et al.*, 2001). In Tokyo, in August 1975, the underground water containing Cr (VI) spoil masses had a 2,000 times higher limit than the permissible limit of chromium (Zayed & Terry, 2003). In India, the chromium level in underground water has been witnessed to be more than 12 mg/L and 550–1,500 ppm/L. The mechanism of ultrastructural organization, biochemical changes and metabolic regulations has not been clarified since the process of phytotoxicity in the aquatic environment by chromium has not been concentrated on in detail (Chandra & Kulshreshtha, 2004). The discharge of industrial wastes and ground water contamination has drastically increased the concentration of chromium in soil (Bielicka *et al.*, 2005). During manufacturing of chromate, the deposit of the Cr residues and waste water irrigation posed a serious Cr pollution to farmland. With the implementation of modern agriculture there is continuous release of Cr into the environment by means of Cr residues, Cr dust and Cr waste water irrigation, resulting in soil pollution affecting the soil-vegetable system and also disturbing the vegetable yield and its quality to humans (Duan *et al.*, 2010). The presence of excess of chromium beyond the permissible limit is destructive to plants since it severely affects the biological factors of the plant and enters the food chain on consumption of these plant materials. Common features due to Cr phytotoxicity are reduction in root growth, leaf chlorosis, inhibition of seed germination and depressed biomass. Chromium toxicity greatly affects the biological processes in various plants such as maize, wheat, barley, cauliflower, citrullus and in vegetables. Chromium toxicity causes chlorosis and necrosis in plants (Ghani, 2011). Enzymes like catalase, peroxidase and cytochrome oxidase with iron as their component are affected by chromium toxicity. The catalase activity stimulated with excess supply of chromium inducing toxicity has been studied with respect to photosynthesis, nitrate reductase activity, protein content in algae and photosynthetic pigments (Nath *et al.*, 2008). Chromium (III) requires a simple diffusion process to enter into the cell and does not depend on any specific membrane carrier. In contrast to Cr(III), Cr(IV) can easily pass through the cell membrane (Chandra & Kulshreshtha, 2004).

#### Mechanism of chromium toxicity

In the environment, trivalent chromium Cr(III) is generally harmless due to its weak membrane permeability. Hexavalent chromium Cr(VI), on the other hand, is more active in penetrating the cell membrane through passages for isoelectric and isostructural anions such as SO_4_^2–^ and HPO_4_^2–^ channels and these chromates are taken up through phagocytosis. Cr(VI) is a strong oxidizing agent and can be reduced to give ephemeral species of pentavalent and tetravalent chromium that are different from that of Cr(III). Stabilization of the pentavelent form is carried out by glutathione and hence intracellular reduction of Cr[VI] is considered a detoxification mechanism when reduction occurs away from the target region. However if intracellular reduction of Cr[VI] occurs near the target site, it may serve to activate Cr. The reactions between Cr(VI) and biological reductants like thiols and ascorbate result in the production of reactive oxygen species such as superoxide ion, hydrogen peroxide, and hydroxyl radical, ultimately leading to oxidative stress in the cell causing damage to DNA and proteins (Stohs & Bagchi, 1995). According to literature surveys, Cr(VI) has been found to be much more dangerous than Cr(III), since Cr(VI) enters the cells more readily than does Cr(III) and is eventually reduced to Cr(III). Because of its mutagenic properties, Cr(VI) is categorized as a group 1 human carcinogen by the International Agency for the Research on Cancer (Dayan & Paine, 2001; Zhang, 2011).

### Aluminium

Aluminium is the third most abundant element found in the earth's crust (Gupta *et al.*, 2013). Aluminium occurs naturally in the air, water and soil. Mining and processing of aluminium elevates its level in the environment (ATSDR, 2010). Recent investigations on environmental toxicology revealed that aluminium may present a major threat for humans, animals and plants in causing many diseases (Barabasz *et al.*, 2002). Many factors, including pH of water and organic matter content, greatly influence the toxicity of aluminium. With decreasing pH its toxicity increases (Jeffrey *et al.*, 1997). The mobilization of toxic aluminium ions, resulting from changes in the pH of soil and water caused by acid rains and increasing acidification of the surrounding atmosphere, has an adverse effect on the environment. This is manifested by the drying of forests, plant poisoning, crop decline or failure, death of aquatic animals, and also by various imbalances in the function of human and animal systems (Barabasz *et al.*, 2002). A pH of surface layer of soil below 5 (pH<5) can lead to soil acidity which is a major concern around the world that affects crop production. Due to aluminium toxicity, the crop production was constrained to 67% of the total acid soil area in the world. Aluminium is one of the most commonly found elements in the earth crust. Due to acid soils (pH<5), silicon gets leached leaving behind aluminium in solid form known as aluminium oxyhydroxides, such as gibbsite and boehmite. These unstable forms of aluminium discharge phytotoxic Al^3+^ well-known as Al (OH)^63+^ in soil (Ermias Abate *et al.*, 2013). The interaction of Al^3+^ with apoplastic, plasma membrane, and symplastic targets leads to toxicity and distracts the physical and cellular processes in plants. The common manifestations are root growth inhibition, cellular modification in leaves, small and dark green leaves, yellowing and death of leaves, chlorosis, purpling and foliar necrosis (Gupta *et al.*, 2013). Aluminium in high concentrations is very toxic for aquatic animals, especially for gill breathing organisms such as fish, causing osmoregulatory failure by destructing the plasma and hemolymph ions. The activity of gill enzyme, essential for the uptake of ions, is inhibited by the monomeric form of aluminium in fish (Rosseland *et al.*, 1990). Living organisms in water, such as seaweeds and crawfish, is also affected by Al toxicity (Bezak-Mazur, 2001). Aluminium has no biological role and is a toxic nonessential metal to microorganisms (Olaniran *et al.*, 2013). Enzymes such as hexokinase, phosphodiesterase, alkalic phosphatase and phosphoxidase are inhibited by aluminium since it has a greater affinity to DNA and RNA. Metabolic pathways in the living organism involving calcium, phosphorous, fluorine and iron metabolism are affected by aluminium. Aluminium has been found to be very harmful to nervous, osseous and hemopoietic cells (Barabasz1 *et al.*, 2002).

#### Mechanism of aluminium toxicity

Aluminium interferes with most physical and cellular processes. The exact mechanism of absorption of aluminium by the gastrointestinal tract is not understood completely. Based on literature surveys, it is difficult to give a proper time period for aluminium toxicity since some symptoms of aluminium toxicity can be detected in seconds and others in minutes after exposure to aluminium (WHO, 1997). Aluminium toxicity probably results from the interaction between aluminium and plasma membrane, apoplastic and symplastic targets (Kochian *et al.*, 2005). In humans Mg^2+^ and Fe^3+^ are replaced by Al^3+^, which causes many disturbances associated with intercellular communication, cellular growth and secretory functions. The changes that are evoked in neurons by aluminium are similar to the degenerative lesions observed in Alzheimer patients. The greatest complications of aluminium toxicity are neurotoxicity effects such as neuronal atrophy in the locus ceruleus, substantia nigra and striatum **(**Filiz & Meral, 2007)**.**

### Iron

Iron is the second most abundant metal on the earth's crust (EPA, 1993). Iron occupies the 26^th^ elemental position in the periodic table. Iron is a most crucial element for growth and survival of almost all living organisms (Valko *et al.*, 2005). It is one of the vital components of organisms like algae and of enzymes such as cytochromes and catalase, as well as of oxygen transporting proteins, such as hemoglobin and myoglobin (Vuori, 1995). Iron is an attractive transition metal for various biological redox processes due to its inter-conversion between ferrous (Fe^2+^) and ferric (Fe^3+^) ions (Phippen *et al.*, 2008). The source of iron in surface water is anthropogenic and is related to mining activities. The production of sulphuric acid and the discharge of ferrous (Fe^2+^) takes place due oxidation of iron pyrites (FeS_2_) that are common in coal seams (Valko *et al.*, 2005). The following equations represent the simplified oxidation reaction for ferrous and ferric iron (Phippen *et al.*, 2008):

$$2\text{Fe}{\text{S}}_{2}+7{\text{O}}_{2}\to 2\text{FeS}{\text{O}}_{4}+{\text{H}}_{2}\text{S}{\text{O}}_{4}\left(\text{ferrous}\right)$$

$$4\text{FeS}{\text{O}}_{4}+{\text{O}}_{2}+10{\text{H}}_{2}\text{O}\to 4\text{Fe}{\left(\text{OH}\right)}_{3}+4{\text{H}}_{2}\text{S}{\text{O}}_{4}\left(\text{ferric}\right)$$

The concentration of dissolved iron in the deep ocean is normally 0.6 nM or 33.5 × 10^−9^ mg/L. In freshwater the concentration is very low with a detection level of 5 μg/L – ICP, whereas in groundwater the concentration of dissolved iron is very high with 20 mg/L (EPA, 1993). In countries like Lithuania, many people have been exposed to elevated levels of iron through drinking water, as the collected groundwater exceeded the permissible limit set by the European Union Directive 98/83/EC on the quality of drinking water (Grazuleviciene *et al.*, 2009). The abundance of species such as periphyton, benthic invertebrates and a fish diversity are greatly affected by the direct and indirect effects of iron contamination (Vuori, 1995). The iron precipitate will cause considerable damage by means of clogging action and hinder the respiration of fishes (EPA, 1993). A study of iron toxicity on aquatic plants, particularly rice, reported that the growth of species of aquatic reed was found to be inhibited by concentration of 1 mg/L total iron (Phippen *et al.*, 2008). Acid soils restrict rice production and together with Zn deficiency cause a macronutrient disorder in wetland rice. The production of lowland rice was greatly affected by high concentrations of reduced iron (Fe^2+^) in the flooded soils. The features of iron toxicity in rice include high uptake of Fe^2+^ by roots, acropetal translocation into leaves, bronzing of rice leaves and yield loss (Becker & Asch, 2005).

#### Mechanism of iron toxicity

A wide range of harmful free radicals are formed when the absorbed iron fails to bind to the protein, which in turn severely affects the concentration of iron in mammalian cells and biological fluids. This circulating unbound iron results in corrosive effect of the gastrointestinal tract and biological fluids. An extremely higher level of iron enters into the body crossing the rate-limiting absorption step and becomes saturated. These free irons penetrate into cells of the heart, liver and brain. Due to the disruption of oxidative phosphorylation by free iron, the ferrous iron is converted to ferric iron that releases hydrogen ions, thus increasing metabolic acidity. The free iron can also lead to lipid peroxidation, which results in severe damage to mitochondria, microsomes and other cellular organelles (Albretsen, 2006). The toxicity of iron on cells has led to iron mediated tissue damage involving cellular oxidizing and reducing mechanisms and their toxicity towards intracellular organelles such as mitochondria and lysosomes. A wide range of free radicals that are believed to cause potential cellular damage are produced by excess intake of iron. The iron produced hydrogen free radicals attack DNA, resulting in cellular damage, mutation and malignant transformations which in turn cause an array of diseases (Grazuleviciene *et al.*, 2009).

## Effects of heavy metals on humans

There are 35 metals that are of concern for us because of residential or occupational exposure, out of which 23 are heavy metals: antimony, arsenic, bismuth, cadmium, cerium, chromium, cobalt, copper, gallium, gold, iron, lead, manganese, mercury, nickel, platinum, silver, tellurium, thallium, tin, uranium, vanadium, and zinc (Mosby *et al.* 1996). These heavy metals are commonly found in the environment and diet. In small amounts they are required for maintaining good health but in larger amounts they can become toxic or dangerous. Heavy metal toxicity can lower energy levels and damage the functioning of the brain, lungs, kidney, liver, blood composition and other important organs. Long-term exposure can lead to gradually progressing physical, muscular, and neurological degenerative processes that imitate diseases such as multiple sclerosis, Parkinson's disease, Alzheimer's disease and muscular dystrophy. Repeated long-term exposure of some metals and their compounds may even cause cancer (Jarup, 2003). The toxicity level of a few heavy metals can be just above the background concentrations that are being present naturally in the environment. Hence thorough knowledge of heavy metals is rather important for allowing to provide proper defensive measures against their excessive contact (Ferner, 2001).

### Arsenic effects

**Figure 5:**
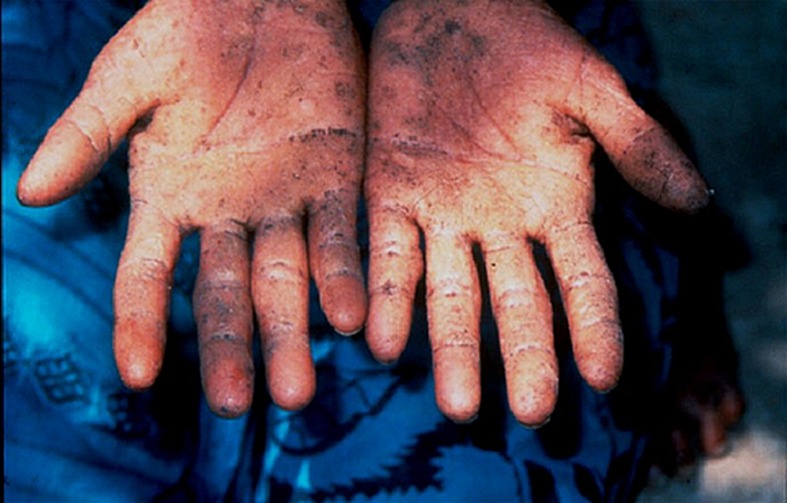
Arsenic keratosis, so called “raindrops on a dusty road” (available from: http://www.pathologyoutlines.com/topic/bonemarrarsenic.html)

**Figure 6:**
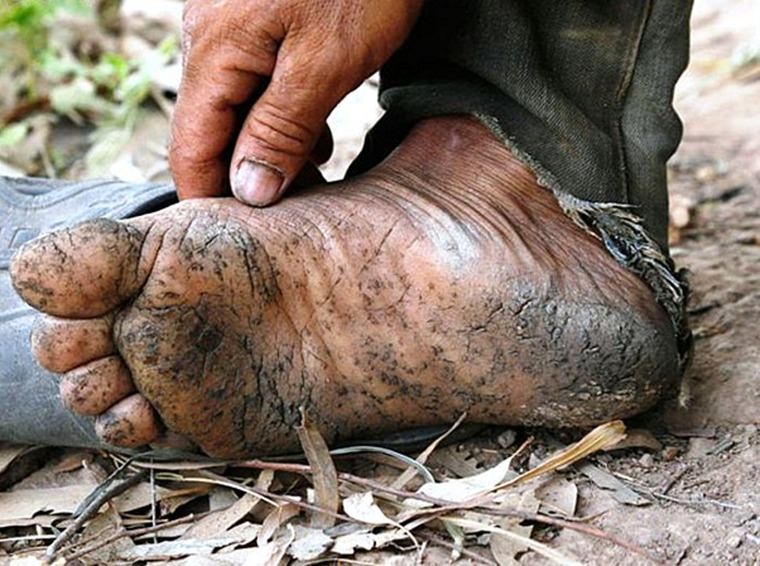
Skin lesions due to arsenicosis (Adapted from Smith *et al.*, 2000).

Arsenic contaminations have occurred as a result of both natural geologic processes and the activities of man. Anthropogenic sources of arsenic include human activities such as mining and processing of ores. The smelting process, both the ancient and a recent one, can release arsenic to the air and soil (Matschullat, 2000). Such types of sources can affect the quality of surface water through groundwater ejection and runoff. Another way of ground water contamination is through geologic sources such as arsenic minerals. The third type of sources are sedimentary and meta-sedimentary bed rocks (Smedley & Kinniburgh, 2002). Most of the paints, dyes, soaps, metals, semi-conductors and drugs contain arsenic. Certain pesticides, fertilizers and animal feeding operations also release arsenic to the environment in higher amounts. The inorganic forms of arsenic such as arsenite and arsenate are found to be more dangerous to human health. They are highly carcinogenic and can cause cancer of lungs, liver, bladder and skin. Humans are exposed to arsenic by means of air, food and water. Drinking water contaminated with arsenic is one of the major causes for arsenic toxicity in more than 30 countries in the world (Chowdhury *et al.*, 2000). If the arsenic level in ground water is 10–100 times the value given in the WHO guideline for drinking water (10 μg/L), it can be a threat to human health (Hoque *et al.*, 2011). Water may get contaminated through improperly disposed arsenical chemicals, arsenical pesticides or by natural mineral deposits. Arsenic toxicity can be either acute or chronic and chronic arsenic toxicity is termed as arsenicosis. Most of the reports of chronic arsenic toxicity in man focus on skin manifestations because of its specificity in diagnosis. Pigmentation and keratosis are the specific skin lesions that indicate chronic arsenic toxicity (Martin & Griswold, 2009). Figure 5 shows arsenic keratosis, so called “raindrops on a dusty road” (Bone marrow – non-neoplastic, benign changes, arsenictoxicity, available from: http://www.pathologyoutlines.com/topic/bonemarrarsenic.html) and Figure 6 shows skin lesions due to arsenicosis (source: Smith *et al.,* 2000).

Lower levels of arsenic exposure can cause nausea and vomiting, reduced production of erythrocytes and leukocytes, abnormal heart beat, pricking sensation in hands and legs, and damage to blood vessels. Long-term exposure can lead to the formation of skin lesions, internal cancers, neurological problems, pulmonary disease, peripheral vascular disease, hypertension and cardiovascular disease and diabetes mellitus (Smith *et al.,* 2000). Chronic arsenicosis results in many irreversible changes in the vital organs and the mortality rate is higher. In spite of the magnitude of this potentially lethal toxicity, there is no effective treatment for this disease (Mazumder, 2008).

### Lead

**Figure 7:**
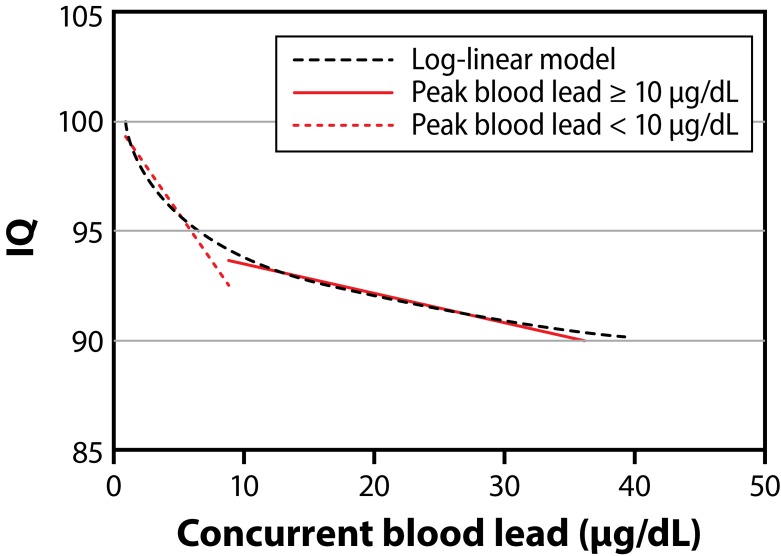
The increase in blood lead concentration affecting a person's IQ (Adapted from Taylor *et al.*, 2012).

**Figure 8:**
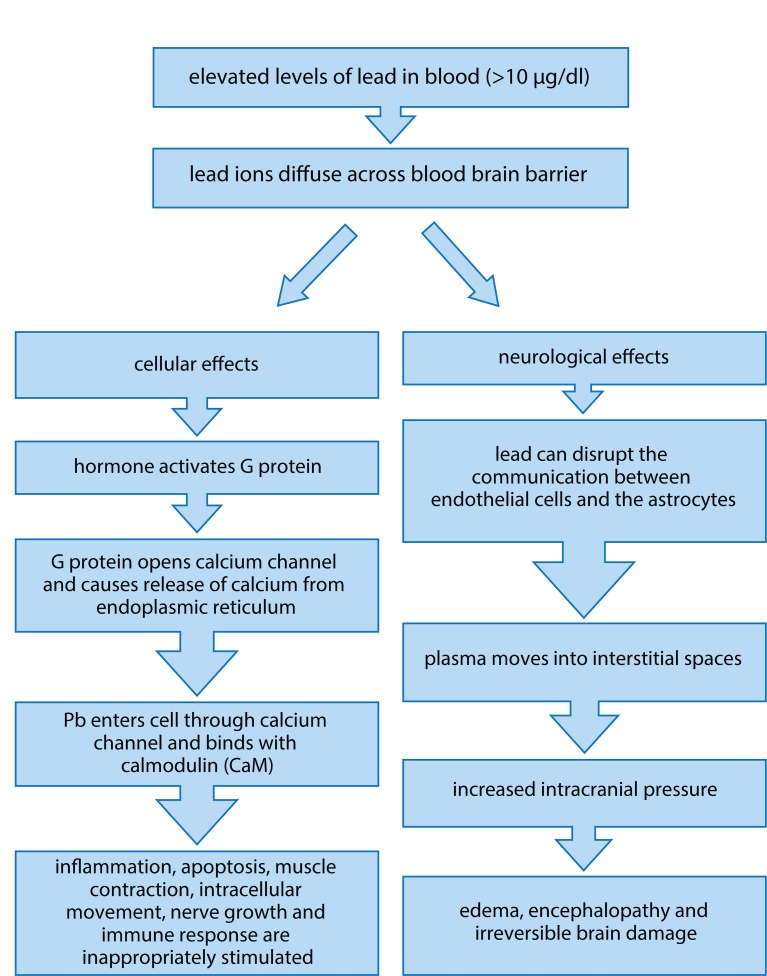
Effects of increased lead level in blood (Adapted from Brochin *et al.,* 2008).

Human activities such as mining, manufacturing and fossil fuel burning has resulted in the accumulation of lead and its compounds in the environment, including air, water and soil. Lead is used for the production of batteries, cosmetics, metal products such as ammunitions, solder and pipes, *etc.* (Martin & Griswold, 2009). Lead is highly toxic and hence its use in various products, such as paints, gasoline, *etc.*, has been considerably reduced nowadays. The main sources of lead exposure are lead based paints, gasoline, cosmetics, toys, household dust, contaminated soil, industrial emissions (Gerhardsson *et al.*, 2002). Lead poisoning was considered to be a classic disease and the signs that were seen in children and adults were mainly pertaining to the central nervous system and the gastrointestinal tract (Markowitz, 2000). Lead poisoning can also occur from drinking water. The pipes that carry the water may be made of lead and its compounds which can contaminate the water (Brochin *et al.*, 2008). According to the Environmental Protection Agency (EPA), lead is considered a carcinogen. Lead has major effects on different parts of the body. Lead distribution in the body initially depends on the blood flow into various tissues and almost 95% of lead is deposited in the form of insoluble phosphate in skeletal bones (Papanikolaou 2005). Toxicity of lead, also called lead poisoning, can be either acute or chronic. Acute exposure can cause loss of appetite, headache, hypertension, abdominal pain, renal dysfunction, fatigue, sleeplessness, arthritis, hallucinations and vertigo. Acute exposure mainly occurs in the place of work and in some manufacturing industries which make use of lead. Chronic exposure of lead can result in mental retardation, birth defects, psychosis, autism, allergies, dyslexia, weight loss, hyperactivity, paralysis, muscular weakness, brain damage, kidney damage and may even cause death (Martin & Griswold, 2009). Figure 7 shows the increase in blood lead concentration affecting a person's IQ (Taylor *et al.*, 2012). Although lead poisoning is preventable it still remains a dangerous disease which can affect most of the organs. The plasma membrane moves into the interstitial spaces of the brain when the blood brain barrier is exposed to elevated levels of lead concentration, resulting in a condition called edema (Teo *et al.* 1997). It disrupts the intracellular second messenger systems and alters the functioning of the central nervous system, whose protection is highly important. Environmental and domestic sources of lead ions are the main cause of the disease but with proper precautionary measures it is possible to reduce the risk associated with lead toxicity (Brochin *et al.*, 2008). Figure 8 shows effects of increased lead level in blood (Brochin *et al.*, 2008).

### Mercury

Mercury is considered the most toxic heavy metal in the environment. Mercury poisoning is referred to as acrodynia or pink disease. Mercury is released into the environment by the activities of various industries such as pharmaceuticals, paper and pulp preservatives, agriculture industry, and chlorine and caustic soda production industry (Morais *et al.*, 2012). Mercury has the ability to combine with other elements and form organic and inorganic mercury. Exposure to elevated levels of metallic, organic and inorganic mercury can damage the brain, kidneys and the developing fetus (Alina *et al.*, 2012). Mercury is present in most foods and beverages in the range <1 to 50 μg/kg. In marine foods it is often seen at higher levels. Organic mercury can easily permeate across the biomembranes and since they are lipophilic in nature, mercury is present in higher concentrations in most species of fatty fish and in the liver of lean fish (Reilly, 2007). Micro-organisms convert the mercury present in soil and water into methyl mercury, a toxin which can accumulate with fish age and with increasing trophic levels. EPA has declared mercuric chloride and methyl mercury to be highly carcinogenic. The nervous system is very sensitive to all types of mercury. Increased exposure of mercury can alter brain functions and lead to shyness, tremors, memory problems, irritability, and changes in vision or hearing. Exposure to metallic mercury vapors at higher levels for shorter periods of time can lead to lung damage, vomiting, diarrhea, nausea, skin rashes, increased heart rate or blood pressure. Symptoms of organic mercury poisoning include depression, memory problems, tremors, fatigue, headache, hair loss, *etc.* Since these symptoms are common also in other conditions, it may be difficult to diagnose such cases (Martin & Griswold, 2009). Due to the excess health effects associated with exposure to mercury, the present standard for drinking water has been set at lower levels of 0.002 mg/L and 0.001 mg/L by the Environmental Protection Act and World Health Organization (WHO, 2004).

**Table 1:** Types of mercuric toxicity.

	Elemental mercury	Methyl mercury	Inorganic mercury
**Sources**	Fossil fuels, dental amalgams, old latex paint, incinerators, thermometers	Pesticides, fish, poultry	Biological oxidation of mercury, demethylation of methyl mercury by intestinal microflora
**Absorption**	75–85% of vapor absorbed	95–100% absorbed in intestinal tract	7–15% of ingested dose absorbed and 2–3% dermal dose absorbed in animals
**Distribution**	Distributed throughout the body, lipophilic, crosses blood-brain barrier and placental barrier, accumulates in brain and kidney	Distributed throughout the body, lipophilic, readily crosses blood-brain barrier as well as placental barrier, accumulates in kidney and brain	Does not cross blood-brain or placental barrier, present in brain neonates, accumulates in kidney
**Excretion**	Sweat, urine, feces, and saliva	90% excreted in bile, feces, 10% in urine	Sweat, saliva, urine and feces
**Reason for toxicity**	Oxidation to inorganic mercury	Demethylation to inorganic mercury, generation of free radical, binding to thiols in enzymes and structural proteins	Binding to thiols in enzymes and structural proteins

Adapted from Patrick, 2002

### Cadmium

**Figure 9:**
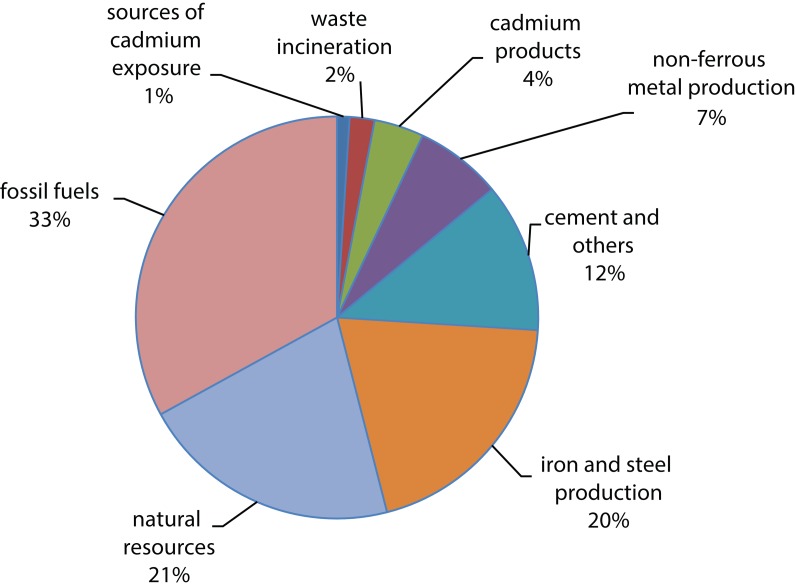
A relative contribution of different sources to human cadmium exposure (Adapted from Regoli, 2005).

**Figure 10:**
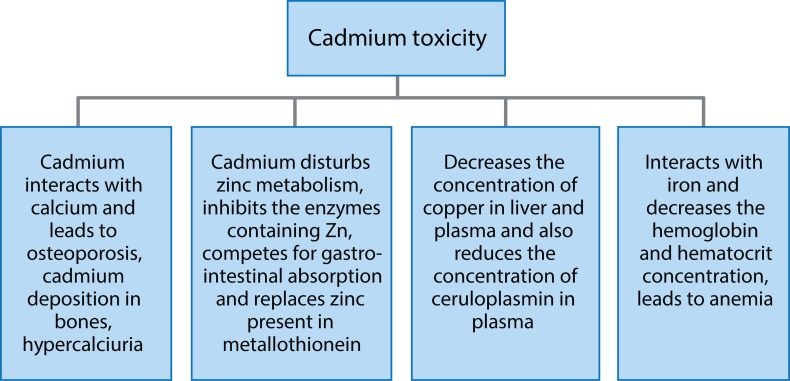
Values of cadmium toxicity (Adapted from Flora *et al.*, 2008).

Cadmium is a metal of the 20^th^ century. It is a byproduct of zinc production. Soils and rocks, including coal and mineral fertilizers, contain some amount of cadmium. Cadmium has many applications, *e.g.* in batteries, pigments, plastics and metal coatings and is widely used in electroplating (Martin & Griswold, 2009). Figure 9 presents a relative contribution of different sources to human cadmium exposure (Regoli, 2005). Cadmium and its compounds are classified as Group 1 carcinogens for humans by the International Agency for Research on Cancer (Henson & Chedrese, 2004). Cadmium is released into the environment through natural activities such as volcanic eruptions, weathering, river transport and some human activities such as mining, smelting, tobacco smoking, incineration of municipal waste, and manufacture of fertilizers. Although cadmium emissions have been noticeably reduced in most industrialized countries, it is a remaining source of fear for workers and people living in the polluted areas. Cadmium can cause both acute and chronic intoxications (Chakraborty *et al.*, 2013). Cadmium is highly toxic to the kidney and it accumulates in the proximal tubular cells in higher concentrations. Cadmium can cause bone mineralization either through bone damage or by renal dysfunction. Studies on humans and animals have revealed that osteoporosis (skeletal damage) is a critical effect of cadmium exposure along with disturbances in calcium metabolism, formation of renal stones and hypercalciuria. Inhaling higher levels of cadmium can cause severe damage to the lungs. If cadmium is ingested in higher amounts, it can lead to stomach irritation and result in vomiting and diarrhea. On very long exposure time at lower concentrations, it can become deposited in the kidney and finally lead to kidney disease, fragile bones and lung damage (Bernard, 2008). Cadmium and its compounds are highly water soluble compared to other metals. Their bioavailability is very high and hence it tends to bioaccumulate. Long-term exposure to cadmium can result in morphopathological changes in the kidneys. Smokers are more susceptible for cadmium intoxication than non-smokers. Tobacco is the main source of cadmium uptake in smokers as tobacco plants, like other plants, can accumulate cadmium from the soil. Non-smokers are exposed to cadmium via food and some other pathways. Yet cadmium uptake through other pathways is much lower (Mudgal *et al.*, 2010). Figure 10 shows values of cadmium toxicity (Flora *et al.*, 2008). Cadmium interacts with essential nutrients through which it causes its toxicity effects. Experimental analysis in animals has shown that 50% of cadmium gets absorbed in the lungs and less in the gastrointestinal tract. Premature birth and reduced birth weights are the issues that arise if cadmium exposure is high during human pregnancy (Henson & Chedrese, 2004).

### Chromium

Chromium is present in rocks, soil, animals and plants. It can be solid, liquid, and in the form of gas. Chromium compounds are very much persistent in water sediments. They can occur in many different states such as divalent, four-valent, five-valent and hexavalent state. Cr(VI) and Cr(III) are the most stable forms and only their relation to human exposure is of high interest (Zhitkovich, 2005). Chromium(VI) compounds, such as calcium chromate, zinc chromates, strontium chromate and lead chromates, are highly toxic and carcinogenic in nature. Chromium (III), on the other hand, is an essential nutritional supplement for animals and humans and has an important role in glucose metabolism. The uptake of hexavalent chromium compounds through the airways and digestive tract is faster than that of trivalent chromium compounds. Occupational sources of chromium include protective metal coatings, metal alloys, magnetic tapes, paint pigments, rubber, cement, paper, wood preservatives, leather tanning and metal plating (Martin & Griswold, 2009). Schroeder *et al.* (1970) reported that cigarettes contained 390 g/kg of Cr, but there has been no significant report published on the amount of chromium inhaled through smoking. When broken skin comes in contact with any type of chromium compounds, a deeply penetrating hole will be formed. Exposure to chromium compounds can result in the formation of ulcers, which will persist for months and heal very slowly. Ulcers on the nasal septum are very common in case of chromate workers. Exposure to higher amounts of chromium compounds in humans can lead to the inhibition of erythrocyte glutathione reductase, which in turn lowers the capacity to reduce methemoglobin to hemoglobin (Koutras *et al.*, 1965; Schlatter & Kissling, 1973). Results obtained from different *in vitro* and *in vivo* experiments have shown that chromate compounds can induce DNA damage in many different ways and can lead to the formation of DNA adducts, chromosomal aberrations, sister chromatid exchanges, alterations in replication and transcription of DNA (O'Brien *et al.*, 2001; Matsumoto *et al.*, 2006).

### Aluminium

Aluminium is the third most common element found on the earth's crust. It exists in only one oxidation state (^3+^) in the environment. The main routes of aluminium consumption by humans are through inhalation, ingestion and dermal contact and sources of exposure are drinking water, food, beverages, and aluminium containing drugs. Aluminium is naturally present in food. Aluminium and its compounds are poorly absorbed in humans, although the rate at which they get absorbed has not been clearly studied. Symptoms that indicate the presence of higher amounts of aluminium in the human body are nausea, mouth ulcers, skin ulcers, skin rashes, vomiting, diarrhea and arthritic pain. These symptoms have however been reported to be mild and short lived (Clayton, 1989). Aluminium exposure is probably a risk factor for the onset of Alzheimer disease (AD) in humans, as hypothesized by the WHO, 1997. Contact dermatitis and irritant dermatitis were seen in persons who were exposed to aluminium in their place of work. Aluminium showed adverse effects on the nervous system and resulted in loss of memory, problems with balance and loss of co-ordination (Krewski *et al.*, 2009). People suffering from kidney diseases find it difficult to eliminate aluminium from the body, resulting in aluminium accumulation in the body leading to bone and brain damage. Some factors that would likely be the reason for the development of aluminium toxicity are life in dusty environments, long-term intravenous nutrition, diminished kidney function, hemodialysis, drinking or ingesting substances that are high in aluminium content, working in an environment that contains high levels of aluminium. Patients undergoing kidney dialysis may get exposed to aluminium present in contaminated dialysates and phosphate binders. Higher levels of aluminium exposure can change the evolution of secondary hyperparathyroidism, leading to other diseases such as aluminium-induced adynamic bone disease and aluminium-induced osteomalacia, both of which are characterized by low-bone remodeling (Andia, 1996). Some of the other complications associated with aluminium toxicity are lung problems, anemia, impaired iron absorption, nervous system problems, *etc.*

### Iron

Iron is the most abundant transition metal in the earth's crust. Biologically it is the most important nutrient for most living creatures as it is the cofactor for many vital proteins and enzymes. Iron mediated reactions support most of the aerobic organisms in their respiration process. If it is not shielded properly, it can catalyze the reactions involving the formation of radicals which can damage biomolecules, cells, tissues and the whole organism. Iron poisoning has always been a topic of interest mainly to pediatricians. Children are highly susceptible to iron toxicity as they are exposed to a maximum of iron-containing products (Albretsen, 2006). Iron toxicosis occurs in four stages. The first stage which occurs after 6 hrs of iron overdose is marked by gastrointestinal effects such as gastro intestinal bleeding, vomiting and diarrhea (Osweiler *et al.*, 1985). The second stage progresses within 6 to 24hrs of overdose and it is considered as the latent period, a period of apparent medical recovery. The third stage occurs between 12 to 96 hrs after the onset of certain clinical symptoms. This stage is characterized by shocks, hypotension, lethargy, tachycardia, hepatic necrosis, metabolic acidosis and sometimes death (Hillman, 2001). The fourth stage occurs within 2–6 weeks of iron overdose. This stage is marked by the formation of gastrointestinal ulcerations and development of strictures. Excess iron uptake is a serious problem in developed and meat eating countries and it increases the risk of cancer. Workers who are highly exposed to asbestos that contains almost 30% of iron are at high risk of asbestosis, which is the second most important cause for lung cancer (Nelson, 1992). It is said that asbestos associated cancer is linked to free radicals. Loose intracellular iron can also promote DNA damage. Iron can initiate cancer mainly by the process of oxidation of DNA molecules (Bhasin *et al.*, (2002). Iron salts such as iron sulfate, iron sulfate monohydrate and iron sulfate heptahydrate are of low acute toxicity when exposure is through oral, dermal and inhalation routes and hence they have been placed in toxicity category 3. Furthermore, iron salts are considered to be safe by the Food and Drug Administration and their toxic effects are very much negligible. Formation of free radicals is the outcome of iron toxicity (Ryan & Aust, 1992). During normal and pathological cell processing, byproducts such as superoxide and hydrogen peroxide are formed, which are considered to be free radicals (Fine, 2000). These free radicals are actually neutralized by enzymes such as superoxide dismutase, catalase and glutathione peroxidase but the superoxide molecule has the ability to release iron from ferritin and that free iron reacts with more and more of superoxide and hydrogen peroxide forming highly toxic free radicals such as hydroxyl radical (McCord, 1998). Hydroxyl radicals are dangerous as they can inactivate certain enzymes, initiate lipid peroxidation, depolymerize polysaccharides and can cause DNA strand breaks. This can sometimes result in cell death (Hershko *et al.,* 1998).

## Conclusion

In this review we reviewed the effects of some heavy metals, *i.e.* arsenic, lead, mercury, cadmium, chromium, aluminium and iron, on the environment and living organisms, mainly human beings. Effective legislation, guidelines and detection of the areas where there are higher levels of heavy metals are necessary. Failure to control the exposure will result in severe complications in the future because of the adverse effects imposed by heavy metals. Occupational exposure to heavy metals can be decreased by engineering solutions. Monitoring the exposure and probable intervention for reducing additional exposure to heavy metals in the environment and in humans can become a momentous step towards prevention. National as well as international co-operation is vital for framing appropriate tactics to prevent heavy metal toxicity.

## References

[CIT0001] Abate E, Hussien S, Laing M, Mengistu F (2013). Aluminium toxicity tolerance in cereals: Mechanisms, genetic control and breeding methods. Afr J Agric Res.

[CIT0002] Agency for Toxic Substances and Disease Registry (2008). Public Health Statement Aluminium.

[CIT0003] Albretsen J (2006). The toxicity of iron, an essential element. Veterinary medicine.

[CIT0004] Alina M, Azrina A, Mohd Yunus AS, Mohd Zakiuddin S, Mohd Izuan Effendi H, Muhammad Rizal R (2012). Heavy metals (mercury, arsenic, cadmium, plumbum) in selected marine fish and shellfish along the Straits of Malacca. Int Food Res J.

[CIT0005] Andia JB (1996). Aluminum toxicity: its relationship with bone and iron metabolism. Nephrol Dial Transplant.

[CIT0006] Ashe WF, Largent EJ, Dutra FR, Hubbard DM, Blackstone M (1953). Behavior of mercury in the animal organism following inhalation. AMA Arch Ind Hyg Occup Med.

[CIT0007] Barabasz W, Albinska D, Jaskowska M, Lipiec J (2002). Ecotoxicology of Aluminium. Pol J Environ Stud.

[CIT0008] Becker M, Asch F (2005). Iron toxicity in rice – conditions and management concepts. J Plant Nutr Soil Sci.

[CIT0009] Bernard A (2008). Cadmium & its adverse effects on human health. Indian J Med Res.

[CIT0010] Bezak-Mazur E, Widiak M, Ciupa T (2001). A speciation analysis of aluminium in the River Silnica. Pol J Environ Stud.

[CIT0011] Bhasin G, Kauser H, Athar M (2002). Iron augments stage-I and stage-II tumor promotion in murine skin. Cancer Lett.

[CIT0012] Bielicka A, Bojanowska I, Wisniewski A (2005). Two Faces of Chromium-Pollutant and Bioelement. Pol J Environ Stud.

[CIT0013] Brochin R, Leone S, Phillips D, Shepard N, Zisa D, Angerio A (2008). The cellular effect of lead poisoning and its clinical picture. GUJHS..

[CIT0014] Castagnetto JM, Hennessy SW, Roberts VA, Getzoff ED, Tainer JA, Pique ME (2002). MDB: the metalloprotein database and browser at the Scripps Research Institute. Nucleic Acids Res.

[CIT0015] Cervantes C, Campos-García J, Devars S, Gutiérrez-Corona F, Loza-Tavera H, Torres-Guzmán JC, Moreno-Sánchez R (2001). Interactions of chromium with microorganisms and plants. FEMS Microbiol Rev.

[CIT0016] Chakraborty S, Dutta AR, Sural S, Gupta D, Sen S (2013). Ailing bones and failing kidneys: a case of chronic cadmium toxicity. Ann Clin Biochem.

[CIT0017] Chandra P, Kulshreshtha K (2004). Chromium accumulation and toxicity in aquatic vascular plants. Botanical Rev.

[CIT0018] Chen CW, Chen CF, Dong CD (2012). Distribution and Accumulation of Mercury in Sediments of Kaohsiung River Mouth, Taiwan. APCBEE Procedia.

[CIT0019] Chowdhury UK, Biswas BK, Chowdhury TR, Samanta G, Mandal BK, Basu GC, Chakraborti D (2000). Groundwater arsenic contamination in Bangladesh and West Bengal, India. Environ Health Perspect.

[CIT0020] Clayton DB (1989). Water pollution at Lowermoore North Cornwall: Report of the Lowermoore incident health advisory committee.

[CIT0021] Duan N, Wang XL, Liu XD, Lin C, Hou J (2010). Effect of anaerobic fermentation residues on a chromium-contaminated soil-vegetable system. Procedia Environmental Sciences.

[CIT0022] Ferner DJ (2001). Toxicity, heavy metals. eMed J.

[CIT0023] Ferrara R, Mazzolai B, Lanzillotta E, Nucaro EA, Pirrone N (2000). Temporal trends in gaseous mercury evasion from the Mediterranean seawaters. Sci Tot Environ.

[CIT0024] Fine JS (2000). Iron poisoning. Curr Probl Pediatr.

[CIT0025] Flora SJS, Mittal M, Mehta A (2008). Heavy metal induced oxidative stress & its possible reversal by chelation therapy. Indian J Med Res.

[CIT0026] Gardner JL, Al-Hamdani SH (1997). Interactive effects of aluminum and humic substances on Salvinia. J Aquat Plant Manage.

[CIT0027] Gerhardsson L, Dahlin L, Knebel R, Schütz A (2002). Blood lead concentration after a shotgun accident. Environ Health Perspect.

[CIT0028] Ghani A (2011). Effect of chromium toxicity on growth, chlorophyll and some mineral nutrients of *Brassica juncea* L. Egyptian Acad J Biol Sci.

[CIT0029] Gordon JJ, Quastel GH (1948). Effect of organic arsenicals on enzyme system. Biochem J.

[CIT0030] Goyer RA (1990). Lead toxicity: from overt to subclinical to subtle health effects. Environ Health Perspect.

[CIT0031] Grazuleviciene R, Nadisauskiene R, Buinauskiene J, Grazulevicius T (2009). Effects of Elevated Levels of Manganese and Iron in Drinking Water on Birth Outcomes. Polish J of Environ Stud.

[CIT0032] Gupta N, Gaurav SS, Kumar A (2013). Molecular Basis of Aluminium Toxicity in Plants: A Review. Am J of Plant Sci.

[CIT0033] Gürkan R, Ulusoy HI, Akçay M (2012). Simultaneous determination of dissolved inorganic chromium species in wastewater/natural waters by surfactant sensitized catalytic kinetic spectrophotometry. Arabian J Chem.

[CIT0034] Haley BE (2005). Mercury toxicity: genetic susceptibility and synergistic effects. Medical Veritas.

[CIT0035] Han JX, Shang Q, Du Y (2009). Effect of environmental cadmium pollution on human health. Health.

[CIT0036] Henson MC, Chedrese PJ (2004). Endocrine disruption by cadmium, a common environmental toxicant with paradoxical effects on reproduction. Exp Biol Med (Maywood).

[CIT0037] Hershko C, Link G, Ioav C (1998). Pathophysiology of iron overload. Ann N Y Acad Sci.

[CIT0038] Hillman RS, Hardman JG, Limbird LE, Gilman AG (2001). Chapter 54. Hematopoietic agents: growth factors, minerals, and vitamins. Goodman & Gilman's The Pharmacological Basis of Therapeutics.

[CIT0039] Hoque MA, Burgess WG, Shamsudduha M, Ahmed KM (2011). Delineating low-arsenic groundwater environments in the Bengal Aquifer System, Bangladesh. Appl Geochem.

[CIT0040] Hughes JP, Polissar L, Van Belle G (1988). Evaluation and synthesis of health effects studies of communities surrounding arsenic producing industries. Int J Epidemiol.

[CIT0041] Irfan M, Hayat S, Ahmad A, Alyemeni MN (2013). Soil cadmium enrichment: Allocation and plant physiological manifestations. Saudi J Biol Sci.

[CIT0042] Järup L (2003). Hazards of heavy metal contamination. Br Med Bull.

[CIT0043] Jaishankar M, Mathew BB, Shah MS, Gowda KRS (2014). Biosorption of Few Heavy Metal Ions Using Agricultural Wastes. Journal of Environment Pollution and Human Health.

[CIT0044] Khlifi R, Hamza-Chaffai A (2010). Head and neck cancer due to heavy metal exposure via tobacco smoking and professional exposure: A review. Toxicol Appl Pharmacol.

[CIT0045] Kochian LV, Piñeros MA, Hoekenga OA (2005). The physiology, genetics and molecular biology of plant aluminum resistance and toxicity. Plant and Soil.

[CIT0046] Koutras GA, Schneider AS, Hattori M, Valentine WN (1965). Studies on chromated erythrocytes. Mechanisms of chromate inhibition of glutathione reductase. Br J Haematol.

[CIT0047] Krewski D, Yokel RA, Nieboer E, Borchelt D, Cohen J, Harry J, Rondeau V (2007). Human health risk assessment for aluminium, aluminium oxide, and aluminium hydroxide. JJ Toxicol Environ Health B Crit Rev..

[CIT0048] Lambert M, Leven BA, Green RM (2000). New methods of cleaning up heavy metal in soils and water. Environmental science and technology briefs for citizens.

[CIT0049] Lamhamdi M, El Galiou O, Bakrim A, Nóvoa-Muñoz JC, Arias-Estévez M, Aarab A, Lafont R (2013). Effect of lead stress on mineral content and growth of wheat (*Triticum aestivum* and spinach (*Spinacia oleracea*) seedlings. Saudi J Biol Sci.

[CIT0050] Markowitz M (2000). Lead Poisoning. Pediatr Rev.

[CIT0051] Martin S, Griswold W (2009). Human health effects of heavy metals. Environmental Science and Technology Briefs for Citizens.

[CIT0052] Mathew BB, Tiwari A, Jatawa SK (2011). Free radicals and antioxidants: A review. Journal of Pharmacy Research.

[CIT0053] Matschullat J (2000). Arsenic in the geosphere – a review. Sci Total Environ.

[CIT0054] Matsumoto ST, Mantovani MS, Malaguttii MIA, Dias AL, Fonseca IC, Marin-Morales MA (2006). Genotoxicity and mutagenicity of water contaminated with tannery effluents, as evaluated by the micronucleus test and comet assay using the fish Oreochromis niloticus and chromosome aberrations in onion root-tips. Genet Mol Biol.

[CIT0055] Mazumder G (2008). Chronic arsenic toxicity & human health. Indian J Med Res.

[CIT0056] McCord JM (1998). Iron, free radicals, and oxidative injury. Semin Hematol.

[CIT0057] Mohanty M, Kumar Patra H (2013). Effect of ionic and chelate assisted hexavalent chromium on mung bean seedlings (Vigna Radiata l. Wilczek. Var k-851) during seedling growth. JSPB.

[CIT0058] Morais S, Costa FG, Pereira ML, Oosthuizen J (2012). Heavy metals and human health. Environmental health – emerging issues and practice.

[CIT0059] Mosby CV, Glanze WD, Anderson KN (1996). Mosby Medical Encyclopedia, The Signet: Revised Edition.

[CIT0060] Mudgal V, Madaan N, Mudgal A, Singh RB, Mishra S (2010). Effect of toxic metals on human health. Open Nutraceuticals J.

[CIT0061] Mutlu A, Lee BK, Park GH, Yu BG, Lee CH (2012). Long-term concentrations of airborne cadmium in metropolitan cities in Korea and potential health risks. Atmos Environ.

[CIT0062] Nagajyoti PC, Lee KD, Sreekanth TVM (2010). Heavy metals, occurrence and toxicity for plants: a review. Environ Chem Lett.

[CIT0063] Najeeb U, Ahmad W, Zia MH, Malik Z, Zhou W (2014). Enhancing the lead phytostabilization in wetland plant *Juncus effusus* L. through somaclonal manipulation and EDTA enrichment. Arab J Chem.

[CIT0064] Nath K, Shyam S, Singh D, Shanna YK (2008). Effect of chromium and tannery effluent toxicity on metabolism and growth in cowpea (Vigna sinensis L. Saviex Hassk) seedling. Res Environ Life Sci.

[CIT0065] Nelson RL (1992). Dietary iron and colorectal cancer risk. Free Radic Biol Med.

[CIT0066] Nriagu JO (1996). History of global metal pollution. Science.

[CIT0067] O'Brien T, Xu J, Patierno SR (2001). Effects of glutathione on chromium-induced DNA crosslinking and DNA polymerase arrest. Molecular Mechanisms of Metal Toxicity and Carcinogenesis.

[CIT0068] Olaniran AO, Balgobind A, Pillay B (2013). Bioavailability of heavy metals in soil: impact on microbial biodegradation of organic compounds and possible improvement strategies. Int J Mol Sci.

[CIT0069] Osweiler GD, Carson TL, Buck WB, Van Gelder GA (1985). Clinical and diagnostic veterinary toxicology.

[CIT0070] Papanikolaou NC, Hatzidaki EG, Belivanis S, Tzanakakis GN, Tsatsakis AM (2005). Lead toxicity update. A brief review. Med Sci Monitor.

[CIT0071] Patrick L (2002). Mercury toxicity and antioxidants: Part 1: role of glutathione and alpha-lipoic acid in the treatment of mercury toxicity. Altern Med Rev.

[CIT0072] Patrick L (2003). Toxic metals and antioxidants: Part II. The role of antioxidants in arsenic and cadmium toxicity. Altern Med Rev.

[CIT0073] Phippen B, Horvath C, Nordin R, Nagpal N (2008). Ambient water quality guidelines for iron: overview.

[CIT0074] Regoli L (2005).

[CIT0075] Reilly C, Szefer P, Nriagu JO (2007). Pollutants in Food – Metals and Metalloids. Mineral Components in Foods.

[CIT0076] Rodríguez MC, Barsanti L, Passarelli V, Evangelista V, Conforti V, Gualtieri P (2007). Effects of chromium on photosynthetic and photoreceptive apparatus of the alga Chlamydomonas reinhardtii. Environ Res.

[CIT0077] Rosseland BO, Eldhuset TD, Staurnes M (1990). Environmental effects of aluminium. Environ Geochem Health.

[CIT0078] Ryan TP, Aust SD (1992). The role of iron in oxygen-mediated toxicities. Crit Rev Toxicol.

[CIT0079] Saha JC, Dikshit AK, Bandyopadhyay M, Saha KC (1999). A review of arsenic poisoning and its effects on human health. Crit Rev Env Sci Technol.

[CIT0080] Satarug S, Garrett SH, Sens MA, Sens DA (2011). Cadmium, environmental exposure, and health outcomes. Ciência & Saúde Coletiva.

[CIT0081] Schlatter C, Kissling U (1973). Acute fatal bichromate poisoning. Beitrage zur Gerichtlichen Medizin.

[CIT0082] Schroeder HA, Nason AP, Tipton IH (1970). Chromium deficiency as a factor in atherosclerosis. J Chron Dis.

[CIT0083] Sharma P, Dubey RS (2005). Lead toxicity in plants. Brazilian Journal of Plant Physiology.

[CIT0084] Singh N, Kumar D, Sahu A (2007). Arsenic in the environment: effects on human health and possible prevention. J Environ Biol.

[CIT0085] Smedley PL, Kinniburgh DG (2002). A review of the source, behavior and distribution of arsenic in natural waters. Appl Geochem.

[CIT0086] Smith AH, Lingas EO, Rahman M (2000). Contamination of drinking-water by arsenic in Bangladesh: a public health emergency. Bull World Health Organ.

[CIT0087] Stohs SJ, Bagchi D (1995). Oxidative mechanisms in the toxicity of metal ions. Free Radic Biol Med.

[CIT0088] Taylor MP, Winder C, Lanphear BP (2012). Eliminating childhood lead toxicity in Australia: a call to lower the intervention level. MJA.

[CIT0089] Teo J, Goh K, Ahuja A, Ng H, Poon W (1997). Intracranial vascular calcifications, glioblastoma multiforme, and lead poisoning. AJNR.

[CIT0090] Thürmer K, Williams E, Reutt-Robey J (2002). Autocatalytic oxidation of lead crystallite surfaces. Science.

[CIT0091] Trasande L, Landrigan PJ, Schechter C (2005). Public health and economic consequences of methyl mercury toxicity to the developing brain. Environ Health Perspect.

[CIT0092] U.S. EPA (1993). Standard Methods for the Examination of Water and Wastewater.

[CIT0093] Valko MMHCM, Morris H, Cronin MTD (2005). Metals, toxicity and oxidative stress. Curr Med Chem.

[CIT0094] Vardar F, Ünal M (2007). Aluminum toxicity and resistance in higher plants. Adv Mol Biol.

[CIT0095] Vuori K-M (1995). Direct and Indirect effects of iron on river eco systems. Annal Zoo Fennici.

[CIT0096] Wadhwa N, Mathew BB, Jatawa S, Tiwari A (2012). Lipid peroxidation: mechanism, models and significance. Int J Curr Sci.

[CIT0097] WHO (1997). Aluminium.

[CIT0098] WHO (2004). http://ftp.fao.org/es/esn/jecfa/jecfa61sc.pdf.

[CIT0099] Wolińska A, Stępniewska Z, Włosek R (2013). The influence of old leather tannery district on chromium contamination of soils, water and plants. Nat Sci.

[CIT0100] Yongsheng W, Qihui L, Qian T (2011). Effect of Pb on growth, accumulation and quality component of tea plant. Procedia Engineering.

[CIT0101] Zayed AM, Terry N (2003). Chromium in the environment: factors affecting biological remediation. Plant Soil.

[CIT0102] Zhitkovich A (2005). Importance of chromium-DNA adducts in mutagenicity and toxicity of chromium (VI). Chem Res Toxicol.

